# Identification of New Inhibitors with Potential Antitumor Activity from Polypeptide Structures via Hierarchical Virtual Screening

**DOI:** 10.3390/molecules24162943

**Published:** 2019-08-14

**Authors:** Elenilze F. B. Ferreira, Luciane B. Silva, Glauber V. Costa, Josivan S. Costa, Mayara A. T. Fujishima, Rozires P. Leão, André L. S. Ferreira, Leonardo B. Federico, Carlos H. T. P. Silva, Joaquín M. C. Rosa, Williams J. C. Macêdo, Cleydson B. R. Santos

**Affiliations:** 1 Laboratory of Organic Chemistry and Biochemistry, University of the State of Amapá, Macapá 68900-070, AP, Brazil; 2 Laboratory of Modeling and Computational Chemistry, Department of Biological and Health Sciences, Federal University of Amapá, Macapá 68902-280, AP, Brazil; 3 Graduate Program of Pharmaceutical Innovation, Federal University of Amapá, Macapá 68902-280, AP, Brazil; 4 Laboratory of Mathematic Modeling, Federal Institute of Education, Science and Technology of Amapá, Macapá 68909-398, AP, Brazil; 5 Computational Laboratory of Pharmaceutical Chemistry, School of Pharmaceutical Sciences of Ribeirão Preto, University of São Paulo, São Paulo 14040-903, Brazil; 6 Department of Pharmaceutical and Organic Chemistry, Faculty of Pharmacy, Campus of Cartuja, University of Granada, 18071 Granada, Spain; 7 Biosanitary Institute of Granada (ibs.GRANADA), University of Granada, 18071 Granada, Spain; 8 Laboratory of Molecular Modeling and Simulation System, Federal Rural University of Amazônia, Rua João Pessoa, 121, Capanema 68700-030, PA, Brazil

**Keywords:** leukemia, α4β1 receptor, pharmacophore, PCA, HCA

## Abstract

Leukemias are neoplasms that affect hematopoietic cells, which are developed by genetic alterations (mutations) that lead to the loss of proliferation control mechanisms (maturation and/or cell death). The α4β1 integrin receptor is a therapeutic target for inflammation, autoimmune diseases and lymphoid tumors. This study was carried out to search through the antagonists-based virtual screening for α4β1 receptor. Initially, seventeen (17) structures were selected (based on the inhibitory activity values, IC_50_) and the structure with the best value was chosen as the pivot. The pharmacophoric pattern was determined from the online PharmaGist server and resulted in a model of score value equal to 97.940 with 15 pharmacophoric characteristics that were statistically evaluated via Pearson correlations, principal component analysis (PCA) and hierarchical clustering analysis (HCA). A refined model generated four pharmacophoric hypotheses totaling 1.478 structures set of Zinc_database. After, the pharmacokinetic, toxicological and biological activity predictions were realized comparing with pivot structure that resulted in five (ZINC72088291, ZINC68842860, ZINC14365931, ZINC09588345 and ZINC91247798) structures with optimal in silico predictions. Therefore, future studies are needed to confirm antitumor potential activity of molecules selected this work with in vitro and in vivo assays.

## 1. Introduction

It is estimated that cancer alone corresponds to 21% of the total amount of deaths registered. For the next 30 years, the World Health Organization (WHO) has estimated 75 million people will be living with cancer. Among the rarer types is leukemia, which occupies the tenth position when it comes to mortality by type of cancer [1].

Leukemias are neoplasms that affect stem cells which are developed by genetic alterations through proliferation control mechanisms (maturation and/or cell death), and can be divided into four main groups: acute myeloid (AML), chronic myeloid (CML), acute lymphoid (ALL) and chronic lymphoid (CLL), being differentiated by some properties such as: cellular origin, evolution and response to therapy [2,3].

Acute lymphoid leukemia results in the excessive production of blasts and lymphoid-type cells of T or B lineage [4,5]. T cells are a type of lymphocyte that grow in the thymus and are responsible for the immunological recognition of pathogens. The extracellular proteins, captured by antigen-presenting cells (APCs), are degraded and resulting peptides bind to T-cell receptors (TCRs) determining the immune response [6,7].

Normal and malignant lymphocytes are recirculating cells, and this process requires the cell to be able to cross the endothelium and migrate within the tissues. Integrins are a family of heterodimeric adhesion receptors that are central to both processes that give it the ability to recognize and respond to extracellular matrix ligands. In a study of integrin functions, Vincent et al. reported integrin function, rather than simple expression, as a determinant of disease behavior in lymphocytic leukemia, using fluorescence activated cell classification (FACS) and immunoprecipitation. It was found that when the endothelium was stimulated, an exceptionally increased interaction with the endothelium was observed in approximately half of the cases studied. In these patients, the neoplastic population expressed α4β1, which conferred the ability to adhere strongly to the stimulated endothelium via α4β1 ligand, vascular cell adhesion molecule 1 (VCAM-1) and concluded that constitutive integrin expression/function, intrinsic activation state of the cell and the ability of cytokines to modify integrin-mediated production are combined to determine the different clinical patterns of disease observed in lymphocytic leukemia [6,7,8].

Peptide antagonist compounds were tested for T cell receptors as it has been proven that they bind preferably to malignant lymphoid cells rather than to normal peripheral lymphocytes. Thus, many unnatural amino acid analogs were incorporated to the ligands in the hope that it would increase their binding affinity to malignant T cells [9]. These peptides have origins from T cells of all types of sources, such as bacteria, viruses, products of cellular metabolism, in addition to proteins and lipids that are inherent or foreign to that cell [10,11,12,13].

Computational medicinal chemistry, especially the antagonists-based virtual screening for α4β1 receptors is a technique that can be used in the search for knowledge on the interaction of peptidic ligands with the action mechanism of acute lymphoid leukemia. The target to α4β1 receptor has no crystallography data deposited in the databases, such as in the Protein Data Bank (see site http://www.rcsb.org/). Such a receptor is important for structure-based virtual screening studies. It can be justified at this moment to carry out studies based only on ligands since we do not have information regarding the main residues of amino acids within the active site, and a better understanding of their 3D positions is needed. Allied to this is the computational strategy for the design of novel ligands-based antagonists—the web server PharmaGist generates 3D pharmacophores hypotheses using a structures set that is known for binding to a common target. The server uses the most active compound set and aligns the others according to their conformation as the first ligand input (most active or crystallographic compound), and therefore it is possible to search for new ligands using pharmacophoric regions as templates from the biological activities of known compounds, generating new pharmacophoric hypotheses [14,15,16,17,18].

The pharmacophoric model has important information on the interactions between receptors and their ligands. The model provides a pharmacophoric features set, which leads us to conclude that the use of pharmacophoric models is an excellent tool to obtain new compounds with the same qualities of the bioactive molecule, according to literature studies [19,20,21].

## 2. Results and Discussion

### 2.1. Selection of the Structures according to Inhibitory Activity (IC_50_)

**Figure 1:**
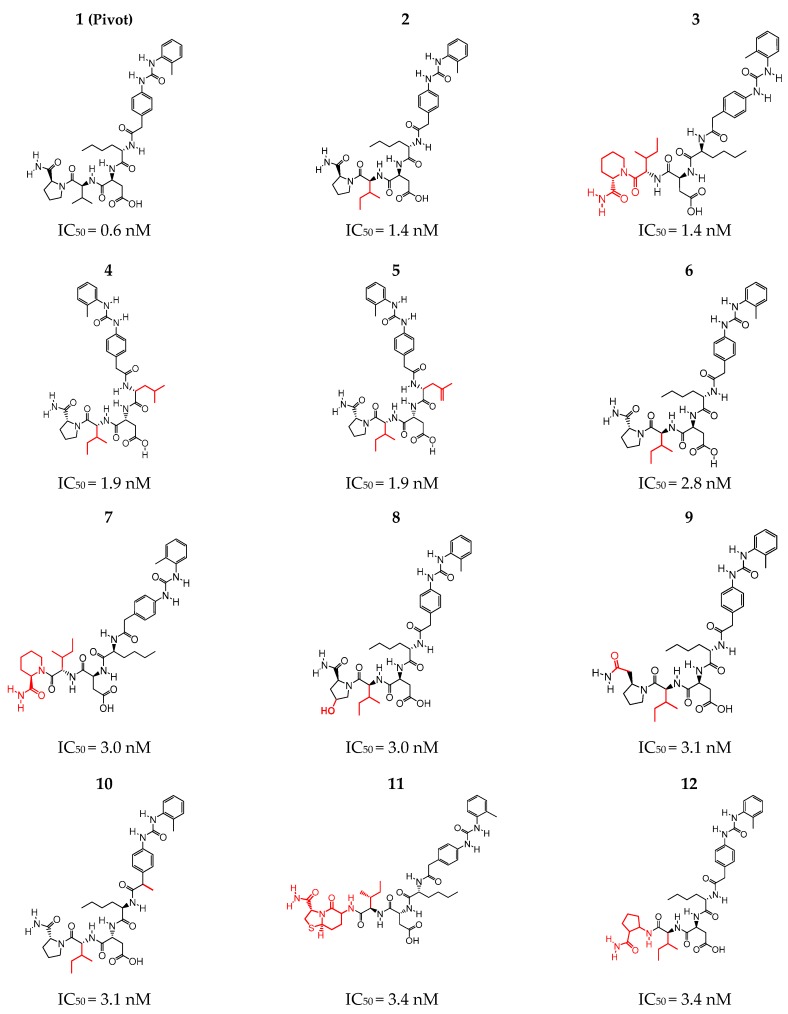

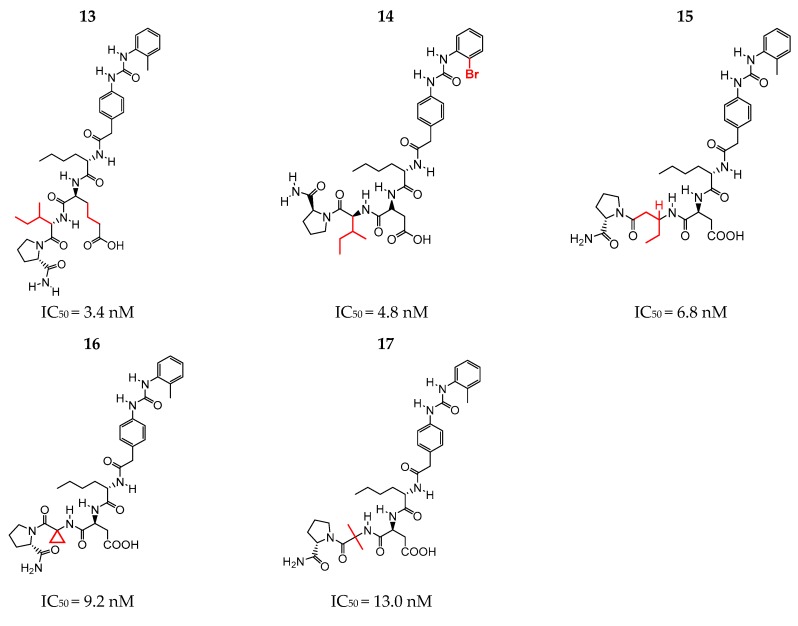
Structures selected (13–17) for the generation of the pharmacophoric hypotheses.

The selection was performed according to the best inhibitory activity values obtained from studies by Lin et al. [12] and Liu et al. [10], where peptidomimetic inhibition assays on Jurkat cell adhesion to immobilized CS-1 and ligand binding affinities were studied in Jurkat cell adhesion assays by inhibiting α4β1-mediated cell adhesion. IC_50_ values indicate the concentration required to inhibit a biological process by half, thus providing a measure of the potency of the antagonist drug [21,22]. The selected structures were organized according to the IC_50_ (nM) values and the structure with the lowest value (IC_50_ = 0.6 nM) was chosen to be the pivot structure (Figure 1).

### 2.2. Optimization of the Geometry of Selected Structures

After selection it was necessary to optimize the structures, since the use of conformations that are not bioactive or pharmacophoric conformations, in conformationally flexible molecules can lead to errors in the interaction models and to solve the problem the structures were redesigned and optimized in the program HyperChem 7.1 [22,23]. The method selected was Molecular Mechanics with the MM^+^ force field [24], which is faster and simpler than the semi-empirical method because the structures in question being polypeptides, have relatively large sizes and thus require time and increase the number of computational cycles required to calculate the energy of the molecule [25]. Molecular mechanics (MM) calculations are force field calculations using gradient lowering methods for geometry optimization, which generally leads to more stable conformation, but not of the least energy. In computational medicinal chemistry, it is considered that of the more stable conformations of the isolated molecule, the bioactive molecule must be present, i.e., the one that binds to the site of action [24,25].

### 2.3. Pharmacophoric Model

#### 2.3.1. Hypothesis Generation

**Figure 2:**
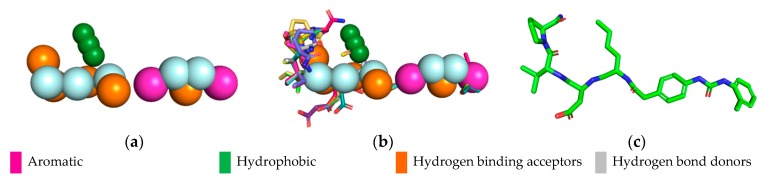
Pharmacophoric model generated with the PharmaGist server. (**a**) Structure 1 (pivot); (**b**) alignment of the 17 structures with better score; (**c**) pharmacophoric features: two aromatic, three hydrophobic, five hydrogen binding acceptors and five hydrogen bond donors. Figure generated with the PyMOL4.5 program.

Obtaining the pharmacophore standard of the selected structures (Figure 2a), using PharmarGist was given by the alignment of Structure 1 (pivot structure) with the other 16 structures, (Figure 2b,c). The aligned structures shared 15 pharmacophoric characteristics: two aromatic (ARO), three hydrophobic (HYD), five hydrogen acceptor (ACC) and five hydrogen donors (DON).

From the data of the model with the score of 97.940, obtained by the PharmaGist server, a matrix was constructed with the pharmacophoric characteristics, atoms (ATM), spatial characteristics (SF), HYD, DON, ACC and ARO (Table 1), which described the individual characteristics of each aligned structure, together with their respective pIC_50_ values (pIC_50_ = −log IC_50_), and Pearson correlation values. The pIC_50_ values were used because, in quantitative structure–activity relationship (QSAR studies), it is frequent to transform the values of their biological activities into their negative logarithmic forms because the more active compounds present higher values, since generally, data of biological activities do not have a normal distribution. The ability to present a great numerical variation in certain intervals is usually solved by transforming each value into its inverse logarithm [24,25,26].

#### 2.3.2. Evaluation of the Pharmacophoric Hypothesis

The pharmacophore characteristics (ATM, SF, HYD, DON, ACC and pIC_50_) were used for the evaluation of the pharmacophoric model by means of statistical methods that could prove the alignment of the structures. The first statistical method used was the Pearson correlation that aimed to show the correlation between the pharmacophoric characteristics and the inhibitory activity of the structures. Along with Pearson’s correlation, the value of *p* was also calculated so that it was possible to evaluate among the correlations which values should be considered in the analysis (Table 1). It is also observed in this table that the correlation between the pairs of pharmacophoric characteristics was less than 0.913, while the correlation between the inhibitory activity (pIC_50_) was less than 0.604. The pharmacophoric characteristics selected represent the characteristics necessary for the generation of pharmacophoric models in the search to identify potential compounds with antileukemic activity.

Principal component analysis (PCA) and hierarchical clustering analysis (HCA) are complementary multivariate statistical techniques that have great acceptance in the analysis of experimental data [25,26]. Statistical methods were used to select the pharmacophoric properties most correlated with biological activity.

PCA was used to evaluate the pharmacophoric data obtained in order to reduce the number of variables and to select the most relevant ones, that is, those responsible for the classification of structures into two groups (more active and less active).

The results of the pharmacophoric model are presented in Table 2. The model was constructed with three main components (3PCs).

The first major component (PC1) described 93.3% of the total information, the second major component (PC2) described 5.0% and the third major component (PC3) described 1.4%. It was observed that PC1 contained 93.3% of the original data and the combination of (PC1 + PC2) 98.3% and (PC1 + PC2 + PC3) accounted for 99.8% of the total information, losing only 0.2% of the original data. The ATM and SF descriptors were the main contributors to PC1, while in PC2 the main contributor was HYD.

The main components can be written as a linear combination of the pharmacophoric characteristics, in terms of the original variables through parameters, given by the components of the eigenvectors. With the values of the eigenvectors it was possible to construct the mathematical expressions (Equations (1) and (2)):

PC1 = 0.882 ATM + 0.370 SF + 0.286 HYD + 0.036 HD + 0.050 HA(1)

PC2 = −0.395 ATM + 0.400 SF + 0.765 HYD − 0.123 HD − 0.290 HA(2)

**Figure 3:**
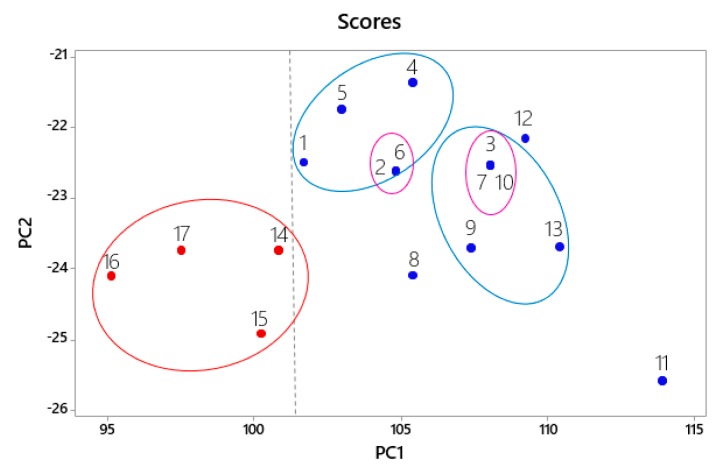
Graphic of the principal components 1 and 2 (PC1–PC2) scores for the most active structures in blue and less active in red.

After obtaining the data and mathematical expressions it was possible to obtain the graph of the two main PCs, which were responsible for most of the variance. Figure 3 shows the scores chart from the analysis of PC1 and PC2.

It is observed in figure the scores of the 17 structures, based on the graph, PC1 distinguishes between the more and less active compounds. The most active compounds are on the right (+1, +2, +3, +4, +5, +6, +7, +8, +9, +10, +11, +12 and +13). while the less active ones are to the left of the graph (−14, −15, −16 and −17).

**Figure 4:**
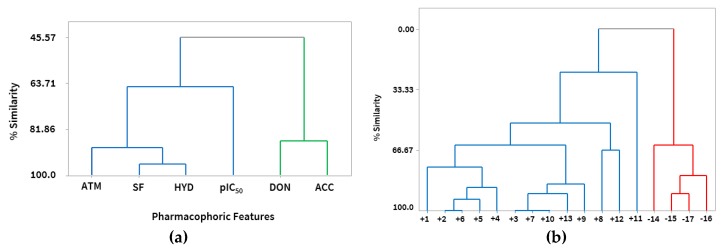
(**a**) Dendrogram of hierarchical clustering analysis (HCA), correlation between pharmacophoric characteristics and pIC_50_. (**b**) Dendrogram (HCA) of structures classified as more active in blue and less active in red.

The HCA showed similar results obtained by PCA. By adopting the Euclidean distance measure, in the Pirouett program, the variables were organized into clusters. In Figure 4a, a dendogram with clusters of pharmacophoric characteristics that are most relevant is presented.

The dendogram obtained by taking into account the pharmacophoric characteristics as the dependent variables, allowed to confirm the correlations already described in the Pearson correlations between hydrophobic (HYD), spatial characteristics (SF), atoms (ATM), hydrogen acceptor group (ACC) and hydrogen donor group (DON) with the inhibitory activity (pIC_50_). The HCA method, as well as PCA, also classified the structures into two classes (more active and less active), according to their similarities, as we can see in Figure 4b.

It was observed that there were similarities between the structures, where it was possible to identify two main clusters. In the largest cluster, called the most active, were the structures from 1 to 13 with the best inhibitory activity values, and in the smaller cluster were the structures from 14 to 17 being the least active, as can be proven both in the dendogram, Figure 4b and in the graph of Figure 3 of the PCA.

It was possible to observe in the group of the most active structures that the similarity between Structures 2 and 6 was in the stereochemistry of carbon 2 of the pyrrolidine group, that in Structure 6 the carbon 2 had an absolute configuration **R** and in Structure 2 the carbon had a **S** configuration, and as a function of this difference the activity in structure 2 (IC_50_ = 1.4 nM) was twice that of structure 6, (IC_50_ = 2.8 nM) (Figure 1).

It can be observed in the dendogram of Figure 4b that Structures 3, 7 and 10 had high similarity. In Structures 3 and 7 the absolute configurations of the stereogenic center of carbon 2 of the piperidinic group were different; Structure 3 had configuration **S** and twice the value of inhibitory activity with respect to Structure 7 where the configuration of the stereogenic center had an absolute configuration **R**.

Between Structures 3, 7 and 10, the structures had similarity in the carbon chain number, but a difference could be observed that may explain the different activities—an additional methyl in Structure 10 of the carbon backbone. The addition of a methyl made the activity of Structure 10 decrease 12% relative to Structure 7 and 29% relative to Structure 3. Increased lipophilicity due to methylation may alter the pharmacological properties and bioavailability and thus the efficacy of a bioactive molecule, such as its mode of interaction with the receptors [27].

### 2.4. Pharmacophoric Hypothesis

The model obtained through PharmaGist was submitted to the ZINCpharmer server [28], to obtain the spatial coordinates of the pharmacophore. The aligned structures shared 15 spatial characteristics (Table 3) generating a model with the following coordinates:

The pharmacophoric model obtained with the 15 characteristics was not sufficient for the virtual screening process, but the structure of the pharmacophore pattern of the pivot structure and the groupings of the structures obtained via PharmaGist, Figure 2a,b, was maintained. Because the results obtained from the Pearson correlation confirm the existence of a correlation between the variables, the alignment of the structures in the more and less active regions was also confirmed by HCA and PCA.

Based on the results analysis and with the objective of increasing the number of structural diversity from the virtual screening strategy, combinations of pharmacophoric patterns were performed. Pharmacophore characteristics were used to construct pharmacophoric hypotheses through different combinations, using Equation (3) [26] shown below:

(3) $$Cp,n=\frac{n!}{p!\left(n-p\right)!}$$

where *C* = number of combinations, *p* = model type (*p* ≠ 0, *p* = 1, *p* = 2, ..., *p* = ∞), and *n* = number of variables for the model.

Considering a total of five variables (pharmacophoric characteristics) by simple combination, without repetition, and after submitting all hypotheses to a new refinement via ZINCpharmer server, four possible pharmacophoric hypotheses were obtained (Table 4) with 1.478 structures selected for subsequent pharmacokinetic toxicological predictions.

### 2.5. Pharmacokinetic Properties Prediction

The four pharmacophoric hypotheses were subjected to predictions of pharmacokinetic properties using the QikProp program. Predictions of the pharmacokinetic properties of the compounds selected from the four hypotheses (Table 5) reduced the number of compounds from 1.478 to 24 compounds.

The #star parameter compared the results obtained with the drug parameters present in the QikProp database. A signal was given when a result was outside the range of 95% of values similar the medicines in the QikProp program. Therefore, the results of the four hypotheses showed that there were no violations of the descriptors analyzed (#star and Lipinski’s Rule 5) [29], indicating significant similarity with commercially available drugs.

The percentage of human oral absorption (HOA%) was considered high; all compounds exhibited values greater than 92.0%. Absorption of drugs in the gastrointestinal tract using Caco-2 and MDCK cells was investigated for values above 500 nm/s which were considered good and below 25 nm/s were bad [30,31]. All compounds showed excellent values.

The hydrophilic/lipophilic characteristics of drugs influence their bioavailability and permeability, and log Po/w values less than five are considered good [32]. The log Po/w was calculated for all compounds obtained in the three hypotheses, and the results obtained ranged from 2.958 ≤ logPo/w ≤ 4.995, for this purpose, all the compounds we investigated were within the limits indicated.

The penetration of the blood–brain barrier is a critical factor, since for a compound that does not have activity in the CNS the values of the partition coefficient of brain/blood QPlogBB, must be less than 1 (C_Brain_/C_Blood_ < 1), and for values greater than 1 suggest an action on the central nervous system [33], that is, negative values indicate a higher concentration of the compound in the blood than in the brain. All the results of the four hypotheses have only negative values, which means low possibility of causing side effects [34]. When evaluating the selected compounds in relation to the CNS descriptor, and according to the classification where −2 is for low penetration capacity in the central nervous system and 2 for high penetration [35]. Again, the four hypotheses presented values that indicated low absorption capacity since none presented values above zero.

### 2.6. Prediction of Toxicological Properties

Toxicological investigation, in silico, was performed using the software DEREK 10.0.2 Nexus [36], and Table 6 shows the results of toxicological predictions for the compounds obtained through virtual screening at the ZINCpharmer database in order to verify toxicity alerts because the presence of toxic groups in the compounds could constitute alerts to be investigated.

Nineteen compounds presented, with respect to the structure–toxicity relationship, some type of alert, such as carcinogenicity, mutagenicity and/or skin sensitization. This analysis provided a criterion for excluding potentially harmful substances still in the selection phase.

**Figure 5:**
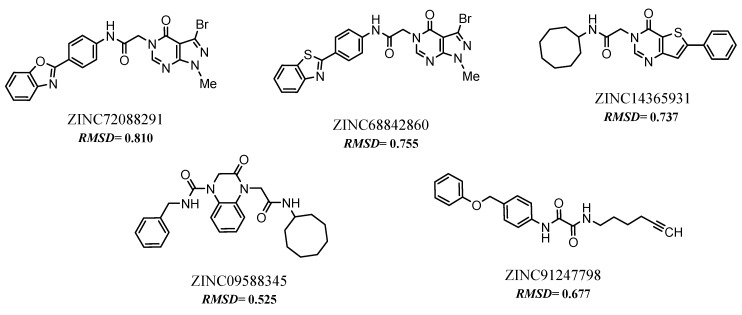
2D Structures and their respective values Root Mean Square Deviation (RMSD).

Using the exclusion criterion mentioned above, only five compounds were selected, since they did not report any toxicity warnings, they were: ZINC72088291, ZINC68842860, ZINC14365931, ZINC09588345 and ZINC91247798, see Figure 5.

### 2.7. Predictions of Biological Activity

After the toxicological prediction, the five selected compounds (see Figure 5) were submitted for prediction of potential biological activity using the Spectral Activity Prediction of the Substances web server (PASS) at http://pharmaexpert.ru/passonline/predict [37]. 

The quantitative parameter obtained tells us that the higher the probability to be revealed (Pa) value in relation to probability of not being revealed (Pi), the greater the probability the molecule under study will have activity on the biological target, therefore activity was considered possible when Pa > Pi [37,38].

For a compound to have cancer-promising activity, it should destroy cancer cells without damaging normal tissues. Recent studies have developed classes of drugs that include: antimetastatic agents, which compromise the surface properties of malignant cells, thus altering their malignant and metastatic potential; biological response modifiers, which alter metabolic and immunological relationships; antineoplastic agents that destroy cancer cells by inhibiting or preventing the growth and proliferation of tumors [38].

The prediction of biological activity of the five compounds obtained in the screening all presented cancer related activities (Table 7) and four of them had prediction values greater than the pivot structure. Predicted/estimated pharmacological activities for these compounds indicated that they were promising for biological activity testing.

### 2.8. Selected Compounds via Pharmacophore-based Virtual Screening

**Figure 6:**
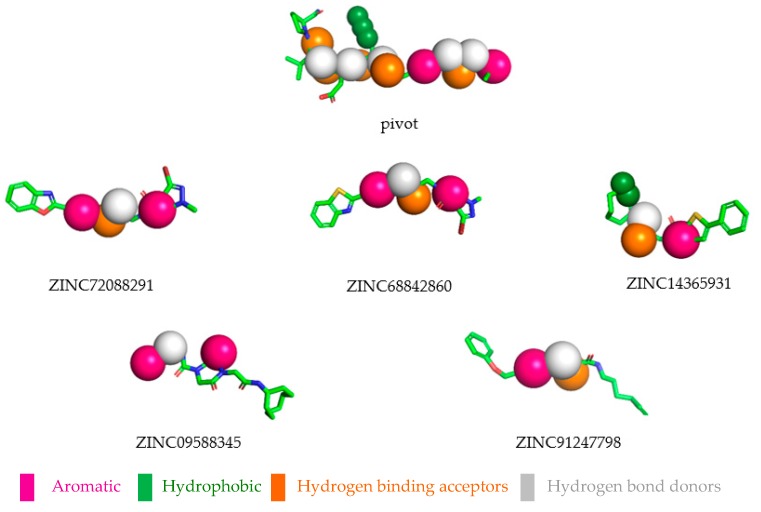
Selected compounds overlapping the pharmacophore.

At the end of the virtual screening process, the five compounds (see Figure 5 and Figure 6) with the most promising results were submitted to a search on SciFinder^®^, available online in the Chemical Abstract Service (CAS) (https://scifinder.cas.org/), to obtain additional information on structures and of the experiments with biological activities. No further information on the compounds selected in the search was found, only information on some physicochemical properties already reported in the ZINCPharmer database. After a search in several databases, no study was found on a possible biological activity for which this research is proposed. The results obtained suggest that the selected compounds can be tested for biological activities with good evidence of reproducing the in silico results. Therefore, future studies are needed to confirm the antitumor potential of these molecules.

## 3. Material and Methods

### 3.1. Selection of Structures

The selection of the structures was performed based on the studies [12,13], where it was proven that a peptide with the D-Pro-Leu-Asp-Ile (PLDI) amino acid sequence bonded through the receptor integrin α4β1, preferably to the lymphoid malignant cells rather than to normal peripheral lymphocytes. Based on this information, amino acid analogues were developed through synthesis with the goal of increasing their binding affinity to malignant T cells [12,13]. Among the 50 compounds obtained from this research, we selected 17 polypeptides using as criteria the values of inhibitory activity (IC_50_). After analyzing the most promising ones, the compound with IC_50_ value of 0.6 nM was chosen as the pivot structure.

### 3.2. Generation of Pharmacophoric Hypotheses

#### 3.2.1. Geometry Optimization

The compounds were designed using the ChemDraw Professional 16.0 software and optimized in the HyperChem 7.1 software [24]. The force field used was MM+ and for this type of calculation the structures must have an initial geometry, therefore the methodology added information on its conformations and geometries from the database of Protein Data Bank (PDB—www.pdb.org) [38] to the optimization of the structures.

#### 3.2.2. Generation of the Pharmacophoric Model

After optimizing the structures, the file was uploaded to the Accelrys Discovery Studio 4.0 software [39]. The structures were overlaid and saved in a single file to submit to the web server PharmaGist (http://bioinfo3d.cs.tau.ac.il/PharmaGist) [16]. The server generated 3D pharmacophores models from the alignment of the pivot structure with the other 16 structures and identified the candidates with the highest score values.

#### 3.2.3. Evaluation of the Pharmacophoric Model

Using the data obtained with the descriptors provided by the PharmaGist result, a matrix with seven descriptors was constructed to make the Pearson correlation, which measures the degree of linear relation. It is a dimensionless quantity that receives a value in the interval from −1 to +1. The coefficient of correlation equal to zero indicates that there is no linear relation between two continuous variables, between 0.2 and 0.4 a weak but existing correlation, between 0.4 and 0.7 a moderate correlation, between 0.7 and 0.9 a strong correlation, and a correlation coefficient of −1 or +1 indicates a perfect linear relation [40,41].

In the analysis, we also considered the significance value for the Pearson correlation coefficient, the value of *p*, where *p* values between 0.05 < *p* ≤ 0.1 show a weak correlation, 0.01 < *p* ≤ 0.05 a strong correlation, and *p* < 0.01 a very strong correlation. This way, it can be easily visualized which variables were related to each other, and it is also possible to compare the relations between different pairs of variables [41,42].

Principal component analysis (PCA) and hierarchical cluster analysis (HCA) were also used with the objective of verifying if the pharmacophoric model that was obtained decreased subjectivity, because they quantify the similarity or dissimilarity between individuals. The Euclidean distance was used for the similarity measurement [25,43]. For the analysis of the HCA and PCA, the statistical software Pirouette 3.0 was used [44].

#### 3.2.4. Pharmacophoric Hypotheses Refinement

The pharmacophoric model obtained had 15 pharmacophoric features, what generated a small number in the search for promising compounds, so the model was submitted again to the ZINCpharmer server for a “refinement” where the pharmacophoric features were maintained, as well as all the coordinates previously obtained. The pharmacophoric model was submitted to ZINCpharmer, the descriptors recombined and thus a larger number of compounds from the chosen database were obtained to increase the number of structural diversities from the virtual strategy.

### 3.3. Pharmacokinetic Predictions

QikProp is fast and accurate software for the prediction of physicochemical properties such as absorption, distribution, metabolism and excretion (ADME) [45]. In addition to predicting molecular properties, QikProp presents comparison ranges between the properties of the molecule or compound being analyzed with 95% of the drugs known and used as reference [45]. 

This approach gives an estimate of the physicochemical properties and bioavailability of the selected compounds, as well as the acceptability of compounds based on the Lipinski’s rule of five [27,29,30,31,32]. According to the Lipinski rule, it is proposed that for a compound to be well absorbed and orally administered, it needs to adapt to the following physicochemical parameters: molecular weight lower than 500 Da, logP (lipophilicity) lower than five; a maximum of five 5 hydrogen donor groups and a maximum of ten hydrogen acceptor groups.

### 3.4. Toxicological Predictions

The toxicity profile of compounds with the best pharmacokinetic profiles was evaluated using the DEREK software [46]. The DEREK (Deductive Estimation of Risk from Existing Knowledge) is a knowledge-based expert system for the qualitative prediction of toxicity. It performs these predictions based on a set of rules, and each rule describes the relationship between a structural attribute or a toxicophoric group and its associated toxicity. Besides carcinogenicity, toxicological points currently covered by the DEREK system also include mutagenicity, skin sensitization, irritation, teratogenicity and neurotoxicity [46,47].

This software makes qualitative predictions and, this way it generates warnings about the possible toxic action of the chemical compounds analyzed by it. The system is able to interpret the toxicophoric substructures present in the compounds as possible inducers of certain types of toxicity, through the correlation rules implemented in the software, operating in two different languages: in the first, which is simpler, it uses of the number of atoms and connections to define the toxicophoric group; in the second, which is more complex, it can answer questions about the structure of the analyzed chemical group [46,47,48].

### 3.5. Prediction of Biological Activity

The activity prediction for a substance is a list of biological activity types for which the probability to be revealed (Pa) and the probability of not being revealed (Pi) are calculated. The values of Pa and Pi are independent, ranging from 0 to 1. The biological activity spectra were predicted for the compounds from the virtual screening with the web server PASS (Prediction of Activity Spectra for Substances) web server [37] and according to studies developed by Ferreira et al. and Ramos et al. [49,50].

## 4. Conclusion

In this study, were applied the computational strategy for the design of novel ligands-based antagonists. The pharmacophoric hypotheses selected have the main characteristics of the ligands that are important in the receptor structure. The chemometric methods of principal component analysis (PCA), hierarchical cluster analysis (HCA), used to confirm the alignment were adequate to confirm this purpose. At the end of the in silico process five compounds with better pharmacokinetic and toxicological properties were selected. The percentage of human oral absorption (HOA%) was considered high, and drug absorption in the gastrointestinal tract using Caco-2 and MDCK cells investigated showed excellent values. None of the compounds selected showed a violation of the Lipinski rule, and did not present any toxicity warnings, and predictions of biological activity all showed predictions of activity related to cancer. The results suggest that they can be tested for biological activities with good evidence of reproducing the results of in silico research. Therefore, future studies are needed to confirm the antitumor potential of these molecules.

## Figures and Tables

**Table 1:** Pharmacophoric characteristics of the training set, pIC_50_ (pIC_50_ = −log IC_50_) values.

Structure	ATM	SF	HYD	DON	ACC	pIC_50_	ARO
1	99	28	11	6	8	9.2219	2
2	102	29	12	6	8	8.8539	2
3	105	30	13	6	8	8.8539	2
4	102	30	13	6	8	8.7212	2
5	100	29	12	6	8	8.5528	2
6	102	29	12	6	8	8.5376	2
7	105	30	13	6	8	8.5229	2
8	103	29	11	7	9	8.5229	2
9	105	29	12	6	8	8.5086	2
10	105	30	13	6	8	8.5086	2
11	111	32	12	7	10	8.4685	2
12	105	31	13	7	8	8.4685	2
13	108	30	13	6	8	8.4685	2
14	99	27	10	6	9	8.3188	2
15	99	26	9	6	8	8.1675	2
16	94	25	8	6	8	8.0362	2
17	96	26	9	6	8	7.8861	2
SF	0.912						
*	0.000						
HYD	0.791	0.913					
*	0.000	0.000					
DON	0445	0.468	0.138				
*	0.074	0.058	0.597				
ACC	0.380	0.278	−0.076	0.649			
*	0.133	0.279	0.771	0.005			
pIC_50_	0.340	0.508	0.604	−0.031	−0.089		
*	0.182	0.037	0.010	0.906	0.735		

* *p* value.

**Table 2:** Main components of the analysis and contribution of pharmacophoric characteristics based on multivariate principal component analysis (PCA).

**Parameters**	**Main Component**			
	**PC1**	**PC2**		**PC3**
**Variance (%)**	93.3	0.05		0.014
**Cumulative variance (%)**	93.3	98.3		99.8
**Pharmacophoric Characteristics**	**Contribution**			
	**PC1**		**PC2**	
**ATM**	0.882		−0.395	
**SF**	0.370		0.400	
**HYD**	0.286		0.765	
**DON**	0.036		−0.123	
**ACC**	0.050		−0.290	

**Table 3:** Spatial coordinates of the pharmacophoric model.

Pharmacophoric Characteristics		Coordinates			
		x	y	z	Radius
Aromatic	Aro 1	12.73	2.75	−0.03	1.1
Aromatic	Aro 2	16.33	9.31	−0.09	1.1
Hydrophobic	Hyd 1	6.95	−1.05	−0.44	1.0
Hydrophobic	Hyd 2	5.68	−0.94	−0.33	1.0
Hydrophobic	Hyd 3	4.41	−0.84	−0.26	1.0
Hydrogen binding acceptors	Acc 1	4.71	−6.08	−1.08	0.5
Hydrogen binding acceptors	Acc 2	7.51	−6.48	−1.85	0.5
Hydrogen binding acceptors	Acc 3	8.96	−3.08	−2.15	0.5
Hydrogen binding acceptors	Acc 4	11.29	−1.10	1.10	0.5
Hydrogen binding acceptors	Acc 5	15.07	5.78	−0.36	0.5
Hydrogen bond donors	Don 1	7.09	−6.68	0.42	0.5
Hydrogen bond donors	Don 2	8.72	−4.06	−0.09	0.5
Hydrogen bond donors	Don 3	10.06	−0.85	−0.83	0.5
Hydrogen bond donors	Don 4	13.0	5.5	0.59	0.5
Hydrogen bond donors	Don 5	14.23	7.61	0.75	0.5

**Table 4:** Pharmacophore hypotheses via ZINCpharmer refinement.

**Hypothesis 1**						
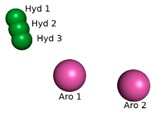	**Pharmacophoric Properties**	**Coordinates**				**Number of Compounds Obtained** **(ZINCpharmer)**
		**x**	**y**	**z**	**R**	
	Aro 1	12.73	2.75	−0.03	1.1	942 structures
	Aro 2	16.33	9.31	−0.09	1.1	
	Hyd 1	5.68	−0.94	−0.33	1.0	
	Hyd 2	6.95	−1.05	−0.44	1.0	
	Hyd 3	4.41	−0.84	−0.26	1.0	
**Hypothesis 2**						
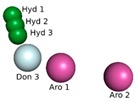	Aro 1	12.73	2.75	−0.03	1.1	141 structures
	Aro 2	16.33	9.31	−0.09	1.1	
	Hyd 1	5.68	−0.94	−0.33	1.0	
	Hyd 2	6.95	−1.05	−0.44	1.0	
	Hyd 3	4.41	−0.84	−0.26	1.0	
	Don 3	10.06	−0.85	−0.83	0.5	
**Hypothesis 3**						
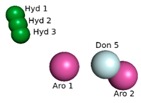	Aro 1	12.73	2.75	−0.03	1.1	9 structures
	Aro 2	16.33	9.31	−0.09	1.1	
	Hyd 1	5.68	−0.94	−0.33	1.0	
	Hyd 2	6.95	−1.05	−0.44	1.0	
	Hyd 3	4.41	−0.84	−0.26	1.0	
	Don 5	14.23	7.61	0.75	0.5	
**Hypothesis 4**						
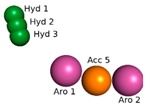	Aro 1	12.73	2.75	−0.03	1.1	386 structures
	Aro 2	16.33	9.31	−0.09	1.1	
	Hyd 1	5.68	−0.94	−0.33	1.0	
	Hyd 2	6.95	−1.05	−0.44	1.0	
	Hyd 3	4.41	−0.84	−0.26	1.0	
	Acc 5	15.07	5.78	−0.36	0.5	

**Table 5:** Pharmacokinetic prediction values for the four hypotheses (QikProp program).

Compounds	RMSD	#star	ROF *	%HOA *	Q_PPCaco_	Q_PPMDCK_	Q_Plog*P*o/w_	CNS *	Q_PlogBB_
**Hypothesis 1**									
pivot	-	10	3	0.0	0.614	1.864	33.047	−2	−4.115
ZINC78538125	0.756	0	0	100	1711.2	884,159	4.383	−1	−0.788
ZINC91247798 **	0.677	0	0	100	1104.2	550,677	4.845	−2	−1.148
ZINC91247798 **	0.718	0	0	100	1104.3	550,732	4.845	−2	−1.148
ZINC32143540	0.756	0	0	100	1819.3	944,695	4.621	−1	−0.811
ZINC78538137	0.792	0	0	100	1182.2	592,819	4.225	−2	−1.121
ZINC02134226	0.749	0	0	100	1153.4	577,244	4.758	−2	−1.042
ZINC14365931	0.737	0	0	100	1003.7	1146.8	3.682	0	−0.456
ZINC01902746	0.749	0	0	100	1153.5	1039.7	4.995	−1	−0.936
ZINC19716136	0.760	0	0	100	717,939	528,751	3.335	−2	−1.005
ZINC09588345 **	0.677	0	0	96.2	567,435	567,156	3.418	−1	−0.792
ZINC09588345 **	0.525	0	0	96.6	592,582	572,285	3.430	−1	−0.789
ZINC64971623	0.528	0	0	100	559,685	557,242	3.629	−1	−0.943
ZINC72088291 **	0.818	0	0	92.6	505,985	594,490	2.959	−1	−0.921
ZINC72088291 **	0.810	0	0	92.8	514,975	605,917	2.958	−1	−0.900
ZINC72088291 **	0.841	0	0	92.8	514,997	605,942	2.958	−1	−0.900
ZINC23592367	0.967	0	0	100	655,789	564,084	4.505	−2	−1.275
ZINC68842860 **	0.803	0	0	96.5	568,385	1055.6	3.467	−1	−0.775
ZINC68842860 **	0.785	0	0	96.5	568,468	1055.8	3.467	−1	−0.774
ZINC68842860 **	0.755	0	0	96.2	555,629	1030.0	3.450	−1	−0.797
ZINC68842860 **	0.827	0	0	100	656,889	1234.4	3.501	−1	−0.711
**Hypothesis 2**									
ZINC91247798 **	0.704	0	0	100	1104.2	550,682	4.845	−2	−1.148
ZINC91247798 **	0.730	0	0	100	1104.3	550,758	4.845	−2	−1.148
**Hypothesis 3**									
ZINC64971623	0.635	0	0	100	559,718	557,260	3.629	−1	−0.943
**Hypothesis 4**									
ZINC68842860	0.772	0	0	96.2	555,605	1030.0	3.450	−1	−0.797

* Rule of five (ROF), human oral absorption (HOA), percentage human oral absorption (%HOA), central nervous system (CNS), the permeability of the differentiated cells of the intestinal epithelium Caco-2 (Q_PPCaco_), Madin–Darby canine kidney (Q_PPMDCK_), the apparent permeability of compound between octanol/water (Q_PlogPo/w_), the apparent permeability of compound in blood–brain barrier (Q_PlogBB_). ** Compound with more than one isomer.

**Table 6:** Toxicity prediction by toxicophoric identification.

Compounds Code	Toxicity Prediction Alert (Lhasa Prediction)	Toxicophoric Group	Toxicity Alert
ZINC78538125	Methaemoglobinaemia	Aniline or precursor	Plausible
	Carcinogenicity	-	Plausible
	Peroxisome proliferation	Alkylaryl or bisaryl, carboxylic acid or precursor	Plausible
ZINC91247798 *	-	-	No alerts
ZINC32143540	Carcinogenicity	-	Plausible
	Peroxisome proliferation	Alkylaryl or bisaryl carboxylic acid or precursor	Plausible
ZINC78538137	Carcinogenicity	-	Plausible
	Peroxisome proliferation	beta-O/S-Substituted carboxylic acid or precursor	Plausible
ZINC02134226	Photoallergenicity	Coumarin	Plausible
	Skin sensitization	Resorcinol or precursor	Plausible
ZINC14365931	-	-	No alerts
ZINC01902746	Photoallergenicity	Coumarin	Plausible
	Skin sensitization	Resorcinol or precursor	Plausible
ZINC19716136	Hepatotoxicity	*p*-Aminophenol or derivative	Plausible
ZINC09588345 *	-	-	No alerts
ZINC64971623	Hepatotoxicity	*p*-Aminophenol or derivative	Plausible
ZINC72088291 *	-	-	No alerts
ZINC23592367	Chromosome damage in vitro	Xanthine	Plausible
	Ocular toxicity	Phosphodiesterase 6 inhibitor and purine base or analogue	Plausible
	Teratogenicity	Xanthine	Plausible
ZINC68842860 *	-	-	No alerts

* Compound with more than one isomer, which showed the same toxicity.

**Table 7:** Prediction of biological activity for compounds resulting from virtual screening.

Compound	Biological Activity	Pa	Pi
pivot	Inhibitor of the cell adhesion molecule	0.629	0.008
	Integrin alpha4 antagonist	0.617	0.002
	Cell adhesion inhibitor	0.558	0.003
	Antineoplastic (Non-Hodgkin’s Lymphoma)	0.442	0.071
	Immunomodulator	0.337	0.052
	Antineoplastic (multiple myelomas)	0.236	0.184
	Integrin alpha4 beta1 antagonist	0.068	0.007
ZINC72088291	Antineoplastic	0.618	0.041
	Immunomodulator	0.337	0.012
ZINC68842860	Antineoplastic	0.638	0.037
	Immunomodulator	0.371	0.037
ZINC14365931	Antineoplastic	0.327	0.042
ZINC09588345	Antimetastatic	0.336	0.067
	Antineoplastic (multiple myelomas)	0.321	0.062
ZINC91247728	Antineoplastic (Lymphocytic Leukemia)	0.447	0.007
	Antineoplastic (Non-Hodgkin’s Lymphoma)	0.388	0.119
	Antileukemic	0.320	0.039
